# Semi-elemental versus polymeric formula for enteral nutrition in brain-injured critically ill patients: a randomized trial

**DOI:** 10.1186/s13054-020-03456-7

**Published:** 2021-01-20

**Authors:** Laurent Carteron, Emmanuel Samain, Hadrien Winiszewski, Gilles Blasco, Anne-Sophie Balon, Camille Gilli, Gael Piton, Gilles Capellier, Sebastien Pili-Floury, Guillaume Besch

**Affiliations:** 1 Department of Anesthesiology and Intensive Care Medicine, University Hospital of Besancon, 3 bvd Alexander Fleming, 25000 Besancon, France; 2 EA3920, University of Franche Comté, Besancon, France; 3 Medical Intensive Care Unit, University Hospital of Besancon, Besancon, France

**Keywords:** Nutrition, Semi-elemental, Brain injury, Critically ill

## Abstract

**Background:**

The properties of semi-elemental enteral nutrition might theoretically improve gastrointestinal tolerance in brain-injured patients, known to suffer gastroparesis. The purpose of this study was to compare the efficacy and tolerance of a semi-elemental versus a polymeric formula for enteral nutrition (EN) in brain-injured critically ill patients.

**Methods:**

Prospective, randomized study including brain-injured adult patients [Glasgow Coma Scale (GCS) ≤ 8] with an expected duration of mechanical ventilation > 48 h. Intervention: an enteral semi-elemental (SE group) or polymeric (P group) formula. EN was started within 36 h after admission to the intensive care unit and was delivered according to a standardized nurse-driven protocol. The primary endpoint was the percentage of patients who received both 60% of the daily energy goal at 3 days and 100% of the daily energy goal at 5 days after inclusion. Tolerance of EN was assessed by the rate of gastroparesis, vomiting and diarrhea.

**Results:**

Respectively, 100 and 95 patients were analyzed in the SE and P groups: Age (57[44–65] versus 55[40–65] years) and GCS (6[3–7] versus 5[3–7]) did not differ between groups. The percentage of patients achieving the primary endpoint was similar (46% and 48%, respectively; relative risk (RR) [95% confidence interval (CI)] = 1.05 (0.78–1.42); *p* = 0.73). The mean daily energy intake was, respectively, 20.2 ± 6.3 versus 21.0 ± 6.5 kcal/kg/day (*p* = 0.42). Protein intakes were 1.3 ± 0.4 versus 1.1 ± 0.3 g/kg/day (*p* < 0.0001). Respectively, 18% versus 12% patients presented gastroparesis (*p* = 0.21), and 16% versus 8% patients suffered from diarrhea (*p* = 0.11). No patient presented vomiting in either group.

**Conclusion:**

Semi-elemental compared to polymeric formula did not improve daily energy intake or gastrointestinal tolerance of enteral nutrition.

***Trial registration*:**

EudraCT/ID-RCB 2012-A00078-35 (registered January 17, 2012).

## Background

Brain-injured critically ill patients admitted to the intensive care unit (ICU) suffer an increased metabolic rate and protein catabolism, leading to a high risk of energy and protein deficits [1–3]. Both are associated with a higher rate of infectious complications, prolonged ICU and hospital length of stay, unfavorable neurological outcome and higher mortality [2, 4]. Early enteral nutrition (EN) is recommended to improve outcome and should be initiated within 48 h after admission to the ICU in hemodynamically stable patients [5]. Despite published guidelines, brain-injured critically ill patients are commonly underfed and receive inadequate intake of both energy and protein [4]. Among the various reasons that have been advanced to explain this is intolerance of EN, related to impaired gastrointestinal mobility, which is common in brain-injured critically ill patients [4]. Indeed, gastroparesis and diarrhea during EN have been reported in, respectively, 20% and 70% of brain-injured critically ill patients [6, 7]. Gastroparesis and diarrhea might alter nutrient absorption and contribute to inadequate energy and protein intake, and could lead physicians to consider interrupting EN [4].

The prescription of a standard isotonic polymeric formula is recommended as the first-choice solution in unselected critically ill patients requiring EN, considering its cost-effectiveness compared to semi-elemental formula [5, 8]. However, semi-elemental solutions containing small peptides and predominantly medium chain triglycerides (MCTs) might theoretically improve gastrointestinal tolerance [9–15]. Proteins hydrolyzed into peptides might facilitate gastric emptying, and the high proportion of MCTs might improve gastrointestinal tolerance and decrease the rate of diarrhea [9, 10, 13–15]. To date, the efficacy and tolerance of semi-elemental formulae have never been specifically addressed in brain-injured critically ill patients.

The hypothesis of the present study was that gastrointestinal tolerance of semi-elemental formula would be better compared to a polymeric formula and would thus improve early energy and protein intake in brain-injured critically ill patients. The aim of the study was therefore to compare the efficacy and tolerance of a semi-elemental versus a polymeric formula in brain-injured critically ill patients.

## Methods

### Study design

We conducted a randomized single-center open-label superiority trial (EudraCT/ID-RCB 2012-A00078-35) in parallel groups, from June 2012 to February 2019 in the medical and surgical intensive care units of the University Hospital of Besancon (Besancon, France). The study protocol was approved by the Institutional Review Board (CPP Est-II, University Hospital of Besancon no. 12/639), and by the French National Health Products Safety Agency (ANSM, Saint-Denis, France no. 2012-A00078-35). The study was conducted in accordance with the French legislation on bioethics [16]. The results are reported in compliance with the Consolidated Standards of Reporting Trials (CONSORT) guidelines.

### Data and endpoint measures

Demographic data, past medical history, Simplified Acute Physiology Score (SAPS) II score and Glasgow Coma Score (GCS) were recorded at admission to the ICU. Gastric residual volume was measured every 12 h. The following data were collected every day during the study period: body weight, sedation (yes/no), catecholamine infusion (yes/no) and the total volume of EN administered. The daily ratio of volume of EN administered to the volume prescribed according to the protocol was calculated.

Blood levels of liver enzymes, albumin and prealbumin were measured at inclusion, at 5 and 10 days after inclusion.

The primary endpoint was the percentage of patients who received both 60% of the daily energy intake goal 3 days after inclusion and 100% of the daily energy intake goal 5 days after inclusion. These thresholds corresponded to the nutritional goals stipulated in published guidelines in force at the time of the study initiation [17, 18].

Secondary endpoints were the tolerance of EN, the nutritional impact of EN, morbidity at 28 days and mortality at 28 and 60 days. Tolerance was assessed by the rate of gastroparesis, vomiting and diarrhea and by the incidence of alteration of blood levels of liver enzymes. Gastroparesis was defined as a gastric residual volume > 500 ml, and diarrhea as more than 3 unusually loose or watery stools per day for 2 consecutive days. Alteration of blood levels of liver enzymes was defined as the occurrence of an abnormal value of aspartate aminotransferase (AST > 34 IU/l) or alanine aminotransferase (ALT > 65 IU/l) (alteration of transaminases), and/or the occurrence of an abnormal value of gamma-glutamyl transferase (GGT > 64 IU/l) (abnormal gamma-glutamyl transferase) during the study period. Nutritional impact was evaluated by the daily energy and protein intakes delivered and on the variation of blood albumin and prealbumin measured at baseline, at Day 5 and at Day 10 after inclusion. Morbidity events considered at 28 days were duration of invasive mechanical ventilation, the length of stay in the ICU and onset of pneumonia. Pneumonia was defined according to the French guidelines by the presence of: fever > 38.3 °C without any other cause, purulent sputum or tracheal aspiration, declining oxygenation or increased oxygen-requirement and new or progressive lung infiltrates on chest radiographs [19]. The reliability of all data collected was assessed by an independent data manager at the end of the study.

### Study population

All consecutive brain-injured critically ill patients admitted with an initial GCS ≤ 8 and an expected duration of mechanical ventilation > 48 h were eligible. Exclusion criteria were: age < 18 years, abdominal surgery during the 14 days prior to inclusion, hemodynamic instability within the 36 first hours in the ICU (defined as an increasing infusion rate of catecholamine, a norepinephrine infusion rate ≥ 3 mg/h or an epinephrine infusion rate ≥ 1 mg/h), contraindication to semi-recumbent position or gastric tube insertion, pregnancy and/or breastfeeding, patient refusal and adults under legal protection. Written informed consent was obtained from a relative prior to inclusion. The deferred consent process was applied if proxies were not contactable at the time of inclusion.

### Randomization and study intervention

Patients included in the study were randomly assigned within 36 h after ICU admission to either the polymeric or the semi-elemental group using a computer-generated randomization list (ratio 1:1; block size of 4). The polymeric group received a hypercaloric (1.5 kcal/ml) polymeric formula (Sondalis HP®, Nestlé Healthcare Science, Vevey, Switzerland), including, per 100 ml, 7.5 g of proteins, 5.8 g of lipids and 17.0 g of carbohydrates (osmolality: 310 mOsm/L). The semi-elemental group received a hypercaloric (1.5 kcal/ml) semi-elemental formula (Peptamen AF®, Nestlé Healthcare Science, Vevey, Switzerland), including, per 100 ml, 9.4 g of proteins hydrolyzed into small peptides, 6.5 g of lipids and 13.5 g of carbohydrates (osmolality: 380 mOsm/L). Ingredients and nutritional content of the EN solutions are detailed in “Appendix 1.” Isocaloric and isovolumic solutions (polymeric or semi-elemental formula) were allocated using opaque envelopes. The solutions allocated by randomization were prescribed for the first 10 days of EN (study period). The investigators were unaware of the randomization block size. Since the packaging and the aspect of the polymeric and semi-elemental solutions were quite different, neither the investigators nor the caregivers were blinded to the treatment allocation. The allocated group was recorded in the patient’s medical file and available to all the caregivers in charge of the patient.

EN was started within 36 h after admission in the ICU at 6 pm in all patients and delivered continuously over 24 h using a pump via a gastric tube. EN was administered following the same standardized protocol in both groups (see “Appendix 2”). This protocol was implemented in the ICU in 2012 and complied with the guidelines in force at the time when the study started [17, 18]. In order to make this nurse-driven protocol easy to implement, the daily energy intake goal was either 1512 or 2268 kcal so that the pump rate was a multiple of 21 ml/h (either 42 ml/h, i.e., 1512 kcal/day in males and females with an estimated ideal body weight using the Lorentz’s formula ≤ 60 kg; or 63 ml/h, i.e., 2268 kcal per day for the others). These two daily caloric intake goals were determined to deliver a daily amount of calories that was as close as possible to the target of 30 kcal/kg of ideal body weight. The initial pump rate was 21 ml/h and was adjusted every 12 h by steps of 21 ml/h according to the gastric residual volume, to reach the nutrition goal. If the gastric residual volume was > 500 ml for more than 12 h, the pump rate was decreased and the EN stopped if necessary. The EN was resumed at a pump rate of 21 ml/h as soon as the gastric residual volume was ≤ 500 ml (see “Appendix 2”). If the patient vomited, EN was stopped for 12 h and resumed at a pump rate of 21 ml/h. Three days of intravenous prokinetics (association of erythromycin 3 mg/kg 3 times per day and metoclopramide 10 mg 3 times per day) were prescribed in case of gastroparesis and/or vomiting. In case of diarrhea, the treatments were: First, 500 ml of normal saline was added to the EN solution for the next 24 h to increase sodium concentration in the digestive tract and the rate of EN was increased according to the protocol described above; if the symptoms persisted, the pump rate was decreased by 21 ml/h for the next 24 h, and then, loperamide 4 mg twice per day was started until disappearance of diarrhea [20]. EN was stopped if the patient needed to be transferred to the operating room, undergo diagnostic investigation out of the ICU or be extubated. EN was then resumed as soon as possible at the same rate as prior to discontinuation. EN was stopped when neurological recovery allowed for withdrawal of invasive mechanical ventilation and resumption of oral feeding. The study period ended when EN was stopped, if the patient was discharged from the ICU, or 10 days after randomization, whichever occurred first. Afterward, patients received standard EN if still required according to the routine protocol used in the ICU. During the study period, patients were sedated with midazolam and sufentanil when necessary. No patient was sedated with propofol.

### Statistical analysis

The sample size calculation was based on a retrospective analysis of data recording sheets from patients admitted to our ICU who received the polymeric formula for EN following the same protocol (data not shown). The expected rate of patients included in the polymeric group who achieved the primary endpoint was 50%. Considering an expected value of the primary endpoint of 70% in the semi-elemental group, at an *α* risk of 0.05 and *β* risk of 0.20, and a loss to follow-up rate of 10%, 103 patients were required in each group.

The Shapiro–Wilk test was used to test the normality of the distribution of quantitative data. Continuous variables are expressed as mean ± standard deviation, or median (interquartile range 25–75%), as appropriate, and categorical variables as number (percentage). Intergroup comparisons were performed using the Chi-square or Fisher’s exact test for qualitative variables and the Student *t* or Mann–Whitney *U* test for quantitative variables, as appropriate. Repeated measurements of daily energy and protein intakes, blood levels of albumin and prealbumin were compared between groups using repeated measure ANOVA. The analysis was neither adjusted nor stratified for additional variables. No subgroup analysis was performed. All statistical analyses were performed with SAS software, version 9.4 (SAS Institute Inc., USA), and the significance level was fixed at 0.05.

## Results

### Study population

**Fig. 1:**
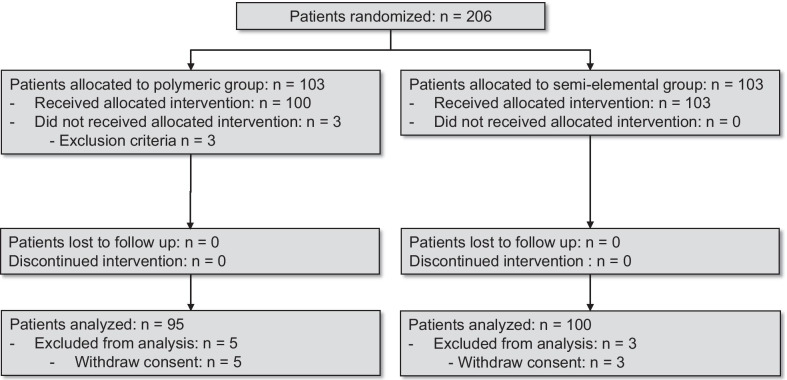
Flowchart of patient inclusions according to the CONSORT (consolidated standards of reporting trials) statement

**Fig. 2:**
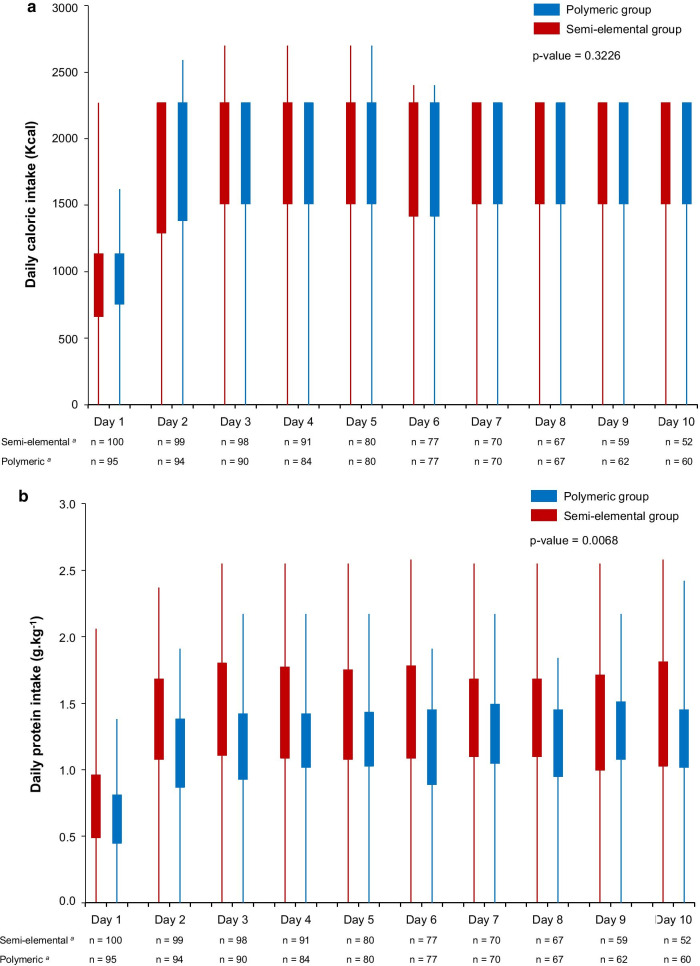
Daily caloric (**a**) and protein (**b**) intake during the study period in the semi-elemental and polymeric groups. Box plots show interquartile range and extreme values. *p* values are from repeated measures ANOVA. ^a^Number of patients still in the study

A total of 206 patients were included during the study period, and 100 and 95 patients were analyzed in the semi-elemental and polymeric groups, respectively. The reasons for exclusion are shown in the study flowchart in Fig. 1. The baseline characteristics of patients were similar between groups (Table 1). The daily caloric intake goals (either 1512 or 2268 kcal per day) and the percentage of goal that was delivered each day in the two groups are presented in “Appendix 3.” The daily energy and protein intake are presented in Fig. 2. Daily protein intake was significantly higher in the semi-elemental group during the study period (p-value for repeated measures ANOVA = 0.0067). The mean daily caloric and protein intake were, respectively, 20.2 ± 6.3 versus 21.0 ± 6.5 kcal/kg/day (mean difference (MD) [95% confidence interval (CI)] = − 0.7 (− 2.6 to 1.1); *p* = 0.42) and 1.3 ± 0.4 versus 1.1 ± 0.3 g/kg/day [95% confidence interval (CI)] = 0.2 (0.1–0.3); *p* < 0.0001) in the semi-elemental and polymeric groups.

**Table 1:** Baseline characteristics of patients in the semi-elemental and polymeric groups

	Semi-elemental group *n* = 100 patients	Polymeric group *n* = 95 patients	*p*
Age (years)	57 [44–65]	55 [40–65]	0.75
Male^a^	67 (67)	53 (56)	0.11
Ideal body weight (kg)	65 [58–70]	64 [56–70]	0.45
Body mass index (kg m^−2^)	26 [23–29]	26 [23–29]	0.63
SAPS II score at admission^*b*^	48 ± 12	49 ± 13	0.52
GCS at admission	6 [3–7]	5 [3–7]	0.15
Type of brain injury^a^			0.50
Traumatic brain injury	49 (49)	46 (48)	
Intracerebral hemorrhage	13 (13)	11 (12)	
Subarachnoid hemorrhage	21 (21)	29 (31)	
Stroke	14 (14)	6 (6)	
Other	3 (3)	3 (3)	
Duration of catecholamine support (days)	1 [0–3]	2 [0–3]	0.72
Duration of sedation (days)	1 [0–3]	2 [0–3]	0.28

Data are median [interquartile range]; ideal body weight was calculated according to the Lorentz formula

*GCS* Glasgow Coma Scale, *SAPS II* Simplified Acute Physiological Score II, *GCS* Glasgow Coma Score

^a^Data are number of patients (percentage)

^b^Data are mean ± standard deviation

### Primary endpoint

Among the 195 patients analyzed, the primary endpoint did not differ significantly between groups (46 (46%) versus 46 (48%) patients, respectively, in the semi-elemental and polymeric groups; [relative risk (RR) (95% CI) = 1.05 (0.78–1.42); *p* = 0.73]). This result was not modified after exclusion of patients in whom EN had been discontinued before Day 3 (7 patients) and Day 5 (28 further patients) after inclusion because of early neurological recovery that enabled resumption of oral feeding. Among the 160 patients who were on EN for at least 5 days, the primary endpoint was met in 46 (58%) versus 46 (57%) patients, respectively, in the semi-elemental and polymeric groups [RR (95% CI) = 0.98 (0.75–1.27); *p* = 0.85]. EN was discontinued for transfer to the operating room or for diagnostic investigation outside of the ICU in 14 (14%) versus 11 (12%) patients in the semi-elemental and polymeric groups, respectively (*p* = 0.61). The median time to administration of 100% of the target volume prescribed according to the protocol after initiation of EN was 1 [1–3] versus 1 [1, 2] days in the semi-elemental and polymeric groups, respectively (*p* = 0.07).

### Secondary endpoints

**Fig. 3:**
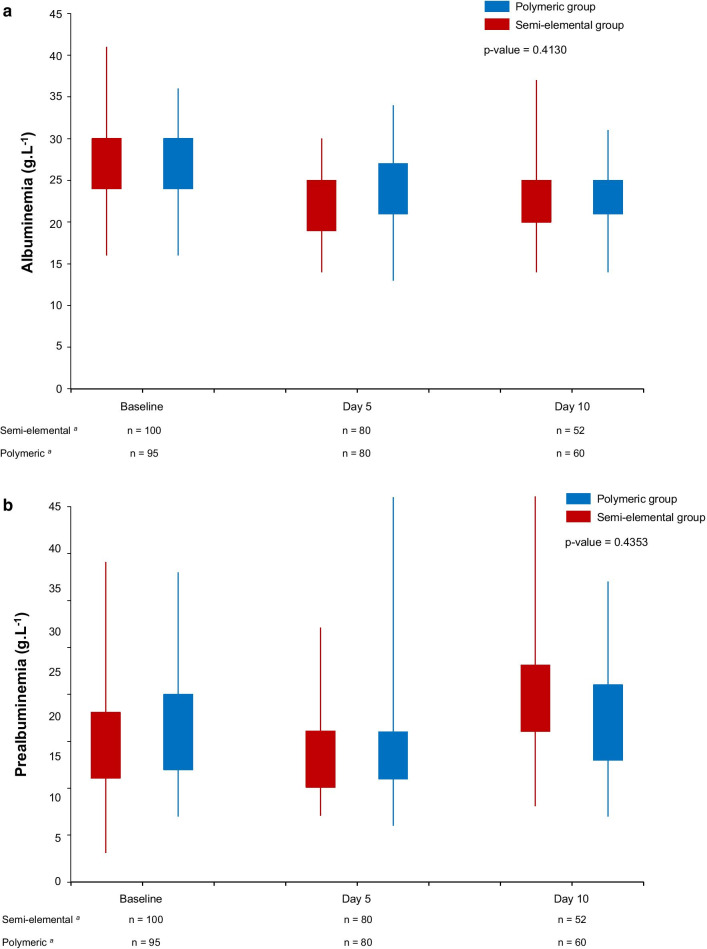
Blood levels of albumin (**a**) and prealbumin (**b**) at baseline, Day 5 and Day 10 after inclusion in the semi-elemental and polymeric groups. Box plots show interquartile range and extreme values. P-values are from repeated measures ANOVA. ^a^Number of patients still in the study

Data on gastrointestinal tolerance, morbidity and mortality are reported in Table 2. The incidence of gastroparesis and diarrhea did not significantly differ between groups (Table 2). No patient in either group presented vomiting during the study period. Figure 3 shows the course of blood albumin and prealbumin over time, and it did not significantly differ between the groups.

**Table 2:** Tolerance of enteral nutrition, morbidity and mortality in the semi-elemental and polymeric groups

Variable	Semi-elemental group *n* = 100 patients		Polymeric group *n* = 95 patients	*p* value
Gastrointestinal tolerance of enteral nutrition				
Gastroparesis	18 (18)		11 (12)	0.21
Requiring interruption of enteral nutrition	6 (6)		5 (5)	1.00
Diarrhea	16 (16)		8 (8)	0.11
Requiring addition of saline^b^	10 (10)		1 (1)	0.01
Requiring loperamide^b^	4 (4)		0 (0)	0.05
Alteration of blood levels of liver enzymes^c^				
Alteration of transaminases	13 (13)		10 (11)	0.66
Abnormal gamma-glutamyl transferase	23 (23)		16 (17)	0.37
Morbidity within 28 days after inclusion				
Length of mechanical ventilation (days)^a^		10 [6–16]	11 [6–17]	0.52
Length of stay in the ICU (days)^a^		14 [8–21]	15 [10–23]	0.18
Pneumonia^d^		47 (47)	41 (43)	0.59
Mortality at 28 days		20 (20)	21 (22)	0.71
Mortality at 60 days		23 (23)	23 (24)	0.81

Data are number of patients (percentage)

*ICU* intensive care unit; gastroparesis was defined as a gastric residual volume > 500 ml, and diarrhea as more than 3 unusually loose or watery stools per day for 2 consecutive days

^a^Data are median [interquartile range]

^b^According to the enteral nutrition protocol, in case of diarrhea, the treatments were: First, 500 ml of normal saline was added to the enteral nutrition solution for the next 24 h; if the symptoms persisted, the pump rate was decreased by 21 ml/h for the next 24 h and then loperamide 4 mg twice per day was started until disappearance of diarrhea

^c^Alteration of transaminases was defined as abnormal value of aspartate aminotransferase (AST > 34 IU/l) or alanine aminotransferase (ALT > 65 IU/l). Abnormal gamma-glutamyl transferase was defined as abnormal value of gamma-glutamyl transferase (GGT > 64 IU/l)

^d^Pneumonia was defined by the presence of: fever > 38.3 °C without any other cause, purulent sputum or tracheal aspiration, declining oxygenation or increased oxygen-requirement, and new or progressive lung infiltrates on chest radiographs [19]

## Discussion

The results of this single-center randomized trial show that semi-elemental formula did not improve gastrointestinal tolerance of EN and early energy intake in brain-injured critically ill patients compared to polymeric formula. Semi-elemental formula increased daily protein intake, but had no impact on blood levels of albumin or prealbumin.

Semi-elemental formulae appear to have no beneficial impact on outcome including gastrointestinal tolerance when prescribed in unselected ICU patients, and were shown to require a decrease of at least 7% in the number of cases of gastrointestinal intolerance in order to be cost-effective in ICU patients [8, 21]. Considering the lack of a clearly demonstrated clinical benefit, and the higher price compared to polymeric formula, the ESPEN guidelines recommend that semi-elemental formulae should not be prescribed as first-line EN solution in ICU patients, but deserve further investigation in patients at high risk of gastrointestinal dysfunction [18]. Brain-injured critically ill patients with intracranial hypertension are at high risk for gastroparesis [4]. Our primary physiological hypothesis was that proteins hydrolyzed into small peptides might promote gastric emptying in this population. Some studies have reported early functional and structural alteration of intestinal mucosa, including villous atrophy, in rat models of traumatic brain injury [22]. Semi-elemental formula has been reported to present nutritional and clinical benefits in nutritionally high-risk non-ICU patients suffering from illnesses that could lead to villous atrophy, such as Crohn’s disease, short bowel syndrome or acute and chronic pancreatitis [9]. The present study is the first randomized controlled trial to specifically address the efficacy and tolerance of semi-elemental EN in brain-injured critically ill patients. Several mechanisms of impaired gastrointestinal function have been identified in this population [23]. This complex pathophysiology might explain the lack of beneficial impact of semi-elemental compared to polymeric formula on gastrointestinal tolerance observed in the present study. The higher fat and protein contents and the higher osmolality in the semi-elemental formula might be another explanation for the lack of difference between the two groups since fat, protein and osmolality could slow gastric emptying and osmolality could promote osmotic diarrhea [24–26].

In terms of advantages, the semi-elemental formula resulted in a significant increase in daily protein delivery compared to the polymeric formula. This increase simply resulted from the higher protein content of the semi-elemental solution. High protein intake might theoretically attenuate ICU-acquired muscle weakness and improve long-term functional outcome by promoting protein synthesis and by preserving muscle mass [27]. Several observational studies have suggested that high protein intake could improve morbidity and mortality [28]. However, the relevance of high protein intake in ICU patients remains controversial, since no beneficial effect and even harmful effects were reported in other observational and randomized controlled studies [12, 29–33]. Nonetheless, the ESPEN guidelines recommend that 1.3 g/kg/day of protein should be gradually delivered in critically ill patients [5]. However, reaching this target remains extremely challenging with standard solutions and requires very-high protein formula to avoid overfeeding while providing high protein intake [12]. Likewise, by promoting gut function and protein absorption, semi-elemental or elemental solutions should be considered in high protein intake strategies in ICU patients [33, 34]. Moreover, beyond the consideration of protein intake, enteral formulae containing 100% of weight-hydrolyzed protein have been reported to have beneficial anti-inflammatory effects in non-ICU elderly patients with acute ischemic stroke [35], whether these anti-inflammatory effects could be observed in brain-injured critically patients, and whether they might improve clinical outcome has never been investigated.

Moreover, our hypothesis was that improved gastrointestinal tolerance would lead to an increase in caloric intake and to improved nutritional status, reflected by higher albumin and prealbumin blood levels. In fact, albumin and prealbumin are negative acute phase proteins and low values might result from a response to inflammation rather than an alteration in nutritional status [36]. This could explain the lack of difference in albumin and prealbumin blood levels between the two groups.

### Limitations of the study

This single-center study was conducted in two independent ICUs, and it is uncertain that the same results would be observed using a different EN protocol. Furthermore, two daily caloric intake goals instead of individual calculations were used. Recent guidelines indeed recommended adjusting daily energy intake to energy expenditure using indirect calorimetry, but this recommendation was applied in only 1% of patients in an international observational study including 1045 brain-injured patients from 341 ICUs [4, 5, 18]. Moreover, the EN protocol used in the present study did not lead to patient underfeeding and about 25% of patients in the two groups were slightly overfed, i.e., had an energy administration between 110 and 120% of the defined target. Nevertheless, the present study aimed to compare the gastrointestinal tolerance of two different enteral nutrition formula rather than to assess the EN protocol. In fact, despite using a different protocol, the incidence of gastroparesis was similar to that reported in previously published studies [4, 6], with a lower rate of patients presenting diarrhea. This could be explained by the heterogeneity in the definition of diarrhea across the different studies. Finally, the primary endpoint was not a hard endpoint such as mortality, as in most previously published randomized controlled trials of nutritional therapy conducted in critically ill patients [37].

## Conclusion

Semi-elemental compared to polymeric formula did not improve gastrointestinal tolerance of enteral nutrition and early caloric intake in brain-injured critically ill patients. These results suggest that standard isotonic polymeric formula might be the first-choice solution in brain-injured critically ill patients requiring EN supports.

## Figures and Tables

**Fig. 4:**
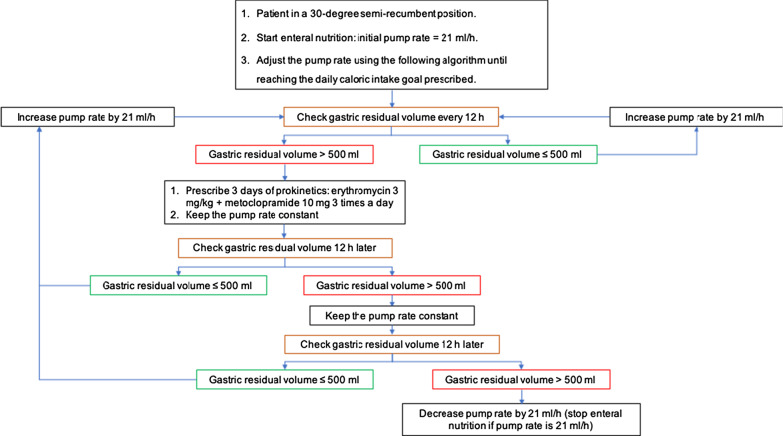
Enteral nutrition protocol flowchart

## Data Availability

The datasets used and/or analyzed during the current study are available from the corresponding author on reasonable request.

## See Table 3.

**Table 3:** Ingredients and nutritional content of Peptamen AF and Sondalis HP

Infusion rate	Peptamen AF		Sondalis HP	
	42 ml/h	63 ml/h	42 ml/h	63 ml/h
Volume (ml)	1008	1512	1008	1512
Osmolality (mOsm/L)	380		310	
Energy (kcal)	1512	2268	1512	2268
Proteins	94.8	142.1	75.6	113.4
Carbohydrates (g)	141.1	211.6	171.4	257.0
of which:				
Sugars	14.1	21.2	19.2	28.7
Fat (g)	65.5	98.3	58.4	87.7
of which:				
Saturates	38.3	57.5	32.3	48.4
Monounsaturates	6.6	9.8	12.1	18.1
Polyunsaturates	10.9	16.3	13.1	19.7
MCT (g)	34.3	51.4	27.2	40.8
Omega-3 (g)	3.6	5.4	2.9	4.4
Omega-6 (g)	8.3	12.4	7.0	10.4
Fibers (g)	0	0	0	0
Salt (g)	2.5	3.8	2.0	3.0
Minerals (mg)				
Sodium (g)	1.0	1.5	0.8	1.2
Chloride (g)	0.8	1.2	1.3	2.0
Potassium (g)	2.3	3.5	1.8	2.7
Calcium (g)	1.0	1.5	0.9	1.4
Phosphorus (g)	0.8	1.3	0.8	1.3
Magnesium (g)	0.3	0.5	0.2	0.3
Iron (mg)	16.1	24.2	12.1	18.1
Zinc (mg)	15.1	22.7	12.1	18.1
Copper (mg)	1.8	2.7	1.2	1.8
Iodine (μg)	201.6	302.4	121.0	181.4
Selenium (μg)	100.8	151.2	60.5	90.7
Manganese (mg)	3.4	5.1	2.0	3.0
Chromium (μg)	75.6	113.4	60.5	90.7
Molybdenum (μg)	181.4	272.2	90.7	136.1
Fluoride (mg)	1.6	2.4	0.9	1.4
Vitamins				
A (μg)	1713.6	2570.4	1038.2	1557.4
D (μg)	17.1	25.7	16.1	24.2
K (μg)	99.8	149.7	83.7	125.5
C (mg)	181.4	272.2	110.9	166.3
B1 (mg)	2.8	4.2	1.7	2.6
B2 (mg)	2.2	3.3	2.2	3.3
B6 (mg)	2.9	4.4	2.5	3.8
Niacin (mg)	12.1	18.1	9.1	13.6
Folic acid (μg)	403.2	604.8	343.7	514.1
B12 (μg)	4.5	6.8	4.0	6.0
Pantothenic acid (mg)	9.5	14.2	7.1	10.6
Biotin (μg)	54.4	81.6	43.3	65.0
E (mg)	29.2	43.8	21,2	31.8

*MCT* medium-chain triglyceride

## See Table 4.

**Table 4:** Daily caloric intake goals and percentage of goal delivered in the semi-elemental and polymeric groups

	Semi-elemental group *n* = 100 patients	Polymeric group *n* = 95 patients
Daily caloric intake goal		
1512 kcal per day	37 (37)	32 (34)
2268 kcal per day	63 (63)	63 (66)
Percentage of daily caloric goal delivered^a^		
Day 1	50 [29–63]	50 [38–50]
Day 2	100 [67–100]	98 [67–100]
Day 3	100 [70–100]	100 [78–100]
Day 4	100 [68–100]	100 [75–100]
Day 5	100 [73–100]	100 [82–100]
Day 6	100 [75–100]	100 [73–100]
Day 7	100 [79–100]	100 [84–100]
Day 8	100 [75–100]	100 [84–100]
Day 9	100 [75–100]	100 [92–100]
Day 10	100 [67–100]	100 [83–100]

Data are number of patients (percentage)

^a^Data are median [interquartile range]

## Abbreviations

EN: Enteral nutrition
GCS: Glasgow Coma Score
GI: Gastrointestinal
ICU: Intensive care unit
MCTs: Medium chain triglycerides
SE: Semi-elemental
